# Picosecond Laser-Ablated Nanoparticles Loaded Filter Paper for SERS-Based Trace Detection of Thiram, 1,3,5-Trinitroperhydro-1,3,5-triazine (RDX), and Nile Blue

**DOI:** 10.3390/nano12132150

**Published:** 2022-06-22

**Authors:** Chandu Byram, Jagannath Rathod, Sree Satya Bharati Moram, Akkanaboina Mangababu, Venugopal Rao Soma

**Affiliations:** 1 Advanced Centre of Research in High Energy Materials (ACRHEM), University of Hyderabad, Hyderabad 500046, India; chandubyram@gmail.com (C.B.); 19acpp04@uohyd.ac.in (J.R.); mssbharathi@uohyd.ac.in (S.S.B.M.); 2 School of Physics, University of Hyderabad, Hyderabad 500046, India; mangababuind@gmail.com

**Keywords:** nanomaterials, flexible substrate, SERS, laser ablation, filter paper, pesticide, thiram, explosive molecules

## Abstract

Recently, filter paper (FP)-based surface-enhanced Raman scattering (SERS) substrates have stimulated significant attention owing to their promising advantages such as being low-cost, easy to handle, and practically suitable for real-field applications in comparison to the solid-based substrates. Herein, a simple and versatile approach of laser-ablation in liquid for the fabrication of silver (Ag)-gold (Au) alloy nanoparticles (NPs). Next, the optimization of flexible base substrate (sandpaper, printing paper, and FP) and the FP the soaking time (5–60 min) was studied. Further, the optimized FP with 30 min-soaked SERS sensors were exploited to detect minuscule concentrations of pesticide (thiram-50 nM), dye (Nile blue-5 nM), and an explosive (RDX-1,3,5-Trinitroperhydro-1,3,5-triazine-100 nM) molecule. Interestingly, a prominent SERS effect was observed from the Au NPs exhibiting satisfactory reproducibility in the SERS signals over ~1 cm^2^ area for all of the molecules inspected with enhancement factors of ~10^5^ and relative standard deviation values of <15%. Furthermore, traces of pesticide residues on the surface of a banana and RDX on the glass slide were swabbed with the optimized FP substrate and successfully recorded the SERS spectra using a portable Raman spectrometer. This signifies the great potential application of such low-cost, flexible substrates in the future real-life fields.

## 1. Introduction

Individual metallic nanoparticles (NPs) have found focused applications in the development of biochemical sensors, photovoltaic energy devices, and surface-enhanced spectroscopy (SERS). Further improvements accomplished by the amalgamation of individual metals within a single nano-entity due to the arising structural changes and compositional complexity [1]. Relative to the pure Au/Ag monometallic NPs, Ag-Au alloy NPs have enthused additional curiosity due to their compositional dependent optical properties such as tunability of localized surface plasmon absorption band (within the limits of individual NPs) and enhanced catalytic properties [2,3,4,5,6]. Most importantly, Ag and Au alloy formation is thermodynamically conceivable and is a feasible process with lower surface segregation as a result of comparable lattice parameters (a_Ag_ = 4.09 Å, a_Au_ = 4.08 Å). Innumerable efforts have been dedicated in the fabrication of Ag-Au alloy NPs including co-reduction of metal salts, evaporation of bulk alloys, galvanic replacement method [2,7,8,9], and laser ablation in liquid (LAL) techniques. In most of the chemical synthesis routes, alloy NPs are obtained in the presence of precursors/artificial surfactants. These surfactants can affect the NPs properties and are also likely to interfere with toxicological assays. Although the removal of residual ligands or surfactants from the NP surfaces is possible by infiltration or centrifugation the whole process is cumbersome. These processes are usually time-consuming and frequently result in inter-particle aggregation [10]. Among the chemical methods described above, LAL is potentially an alternative technique due to its simplicity. This technique is proficient for the simultaneous synthesis of alloy nanomaterials (NPs and NSs) in a single experiment [11,12,13]. Furthermore, large yields (typically few grams/hour can be easily accomplished) of NPs can be obtained using this approach [14,15,16]. A few notable advantages of the LAL method over chemical routes are ease of preparation and providing contamination-free NPs without the necessity of any additional chemicals [17]. In recent years, laser-synthesized nanomaterials have aroused prominent interest in SERS applications due to their modest fabrication procedures in achieving high purity NPs as well as an effortless method for altering the structures (NPs/NSs) sizes and morphology by tuning the laser parameters and surrounding media. Most of the reported laser-fabricated SERS substrates were accomplished by either decorating NPs onto the plain solid substrates, including glass, silicon, and metals, or patterned surface structures followed by analyte deposition. However, solid/patterned-based SERS substrates are slightly costly in nature, difficult to handle (analyte needs to be taken to the substrate), and need further optimization studies before they can be used for real-field applications even though they offer higher enhancement factors, superior sensitivity as well as virtuous reproducibility [18,19,20]. Wu et al. [21] have recently reported the utilization of the tunable scaffold nanoporous silicon embedded with Au NPs as SERS substrate for the detection of pM concentration of methylene blue with the EF ~10^9^. Recently, paper-based SERS substrates have grabbed more attention due to their promising advantages over solid-based substrates, such as (a) low-cost (b) easy to handle (c) practically more suitable for on-field applications [22,23,24,25,26,27]. This is due to the fact that one can collect the analyte molecule from any uneven surface also whereas in the case of solid substrates one need to take the analyte molecule to the substrate. The present work describes the results from the fabrication and characterization of Ag, Au and Ag-Au NPs obtained through the laser ablation of Ag-Ag bulk targets immersed in liquid using ~30 ps laser pulses. Further, these NPs are loaded onto the filter-paper and, subsequently, utilized as SERS platforms for the detection of diverse analytes such as thiram, RDX (1,3,5-Trinitroperhydro-1,3,5-triazine), and Nile blue (NB) in conjunction with a low-cost portable Raman spectrometer. These flexible FP-based SERS substrates demonstrated good enhancement factors in the order of ~10^5^ and relatively lower RSD values of <15% for the measurements performed in the lab and in real-time application.

## 2. Materials and Methods

### 2.1. Materials

Whatman Grade 1 FP with 11 μM porosity and 180 μM thickness; thiram (C_6_H_12_N_2_S_4_), Nile blue (C_20_H_20_ClN_3_O) were purchased from M/s Sigma-Aldrich. RDX (C_3_H_6_N_6_O_6_) was provided by HEMRL (Pune, India). Pure Ag, Ag_70_Au_30_, Ag_50_Au_50_, Ag_30_Au_70_, and Au bulk targets possessing dimensions of 1 × 1 × 0.1 cm^3^ with a purity of ~99.9% were commercially purchased. All reagents used in this study are analytically pure (99.9%). All of the glassware used for the ablation process and storage of the colloidal NP solutions were cleaned/rinsed thoroughly with acetone for three times prior to the laser ablation.

### 2.2. Synthesis of NPs-Laser Ablation

Ag-Au alloy nanoparticles (NPs) were prepared via laser ablation in liquids [28,29,30] using a ps laser delivering pulses at 1064 nm with a duration of ~30 ps and a repetition rate of 10 Hz [31]. For the generation of colloidal solutions, the bulk target was placed at the bottom of a glass vessel filled with 5 mL of distilled water (DW) and the input laser beam was vertically focused on the above-mentioned targets with a plano-convex lens having a focal length of 15 cm. Typically, the target surface was at a depth of ~7 mm from the surface of the DW. The whole setup was mounted on the X-Y motorized stage in order to achieve the raster scanning pattern on the Ag-Au strip during the ablation process. The experiments were employed at constant pulse energy of 15 mJ (fluence of ~47.7 J/cm^2^) and the assessed spot size onto the target surface is ~200 µm. During the ablation, the stages were moved with a scanning speed of 0.2 mm/s (in both X and Y directions) with a line spacing of 60 µm. The raster patterned area on the Ag-Au targets was about 5 × 4 mm^2^. The input laser energy was controlled by adjusting the amplification controller, which was interconnected to the laser source. After the ablation, colloidal solutions were collected and stored in air-tightened glass vials to avoid oxidation due to surrounding atmosphere.

### 2.3. Characterization Techniques

The optical absorption measurements of as-synthesized pure and alloy NPs were achieved by UV-Visible absorption spectrometer (JASCO V-670, Pfungstadt, Germany) in the wavelength regime of 250–1000 nm. Transmission electron microscopy (TEM, FE-Technai G2, Beijing, China, system operated at an accelerating voltage of 300 kV) was utilized to derive the morphological information of as-synthesized NPs such as size, shape, and crystallinity. Further, the morphology of filter paper-soaked with NPs along with their compositions was inspected by a field emission scanning electron microscope (FESEM, Carl ZEISS, Jena, Germany) equipped with an energy dispersive x-ray spectroscope (EDX).

### 2.4. Preparation of SERS Substrates and Measurements

Commercially purchased Whatman filter paper was cut into rectangular strips, soaked in the fabricated NPs about 30 min duration and then used as SERS sensors. The time of soaking was optimized by studying the SERS signal as a function of soaking time. To avoid ambiguity, filter paper (FP) soaked with Ag, Ag_70_Au_30_, Ag_50_Au_50_, Ag_30_Au_70_, and Au are labelled as FPAg, FPAg_70_Au_30_, FPAg_50_Au_50_, FPAg_30_Au_70,_ and FPAu, respectively. The target analytes thiram, Nile blue (NB), and RDX were prepared in-stock solutions by diluting them in methanol. Further, lower concentrations (from 0.1 M to 5 nM) of each analyte were achieved by successive dilution. Afterward, the NPs-soaked filter paper was cut into small pieces (typical sizes ~1 cm^2^), and then an analyte molecule of ~3 µL quantity was dropped over the substrates for detection. The SERS capability of as-fabricated NPs-soaked filter paper substrates was evaluated by a portable-Raman spectrometer (B&W Tek, Township, NJ, USA) with the excitation wavelength of 785 nm. All of the spectra were recorded at 10 mW laser power, spectrum collection time of 5 s, and three (3) accumulations. The spot size of the laser beam on the sample was estimated to be ~100 µm. All of the recorded SERS spectra shown in this work were baseline corrected using software.

## 3. Results and Discussions

**Figure 1:**
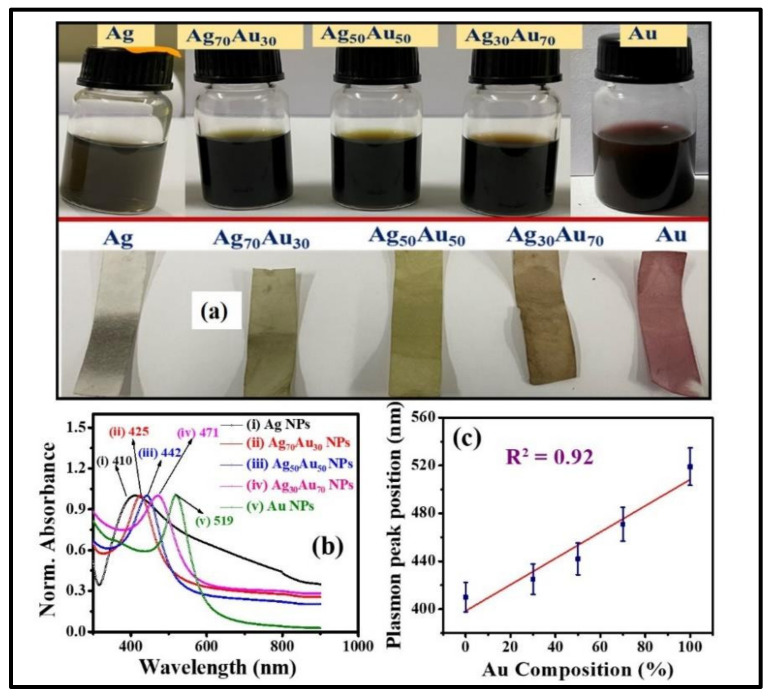
(**a**) Photographic images of as-fabricated Ag-Au alloy NPs including pure Ag and Au NPs (top) and FP soaked with NPs (bottom) (**b**) Normalized UV-Visible absorption spectra of Au-Ag NPs (i) Pure Ag (black) (ii) Ag_70_Au_30_ (red) (iii) Ag_50_Au_50_ (blue) (iv) Ag_30_Au_70_ (pink) and (v) pure Au (green one) (**c**) variation of SPR peak position with increasing Au percentage in Ag-Au alloy NPs.

**Figure 2:**
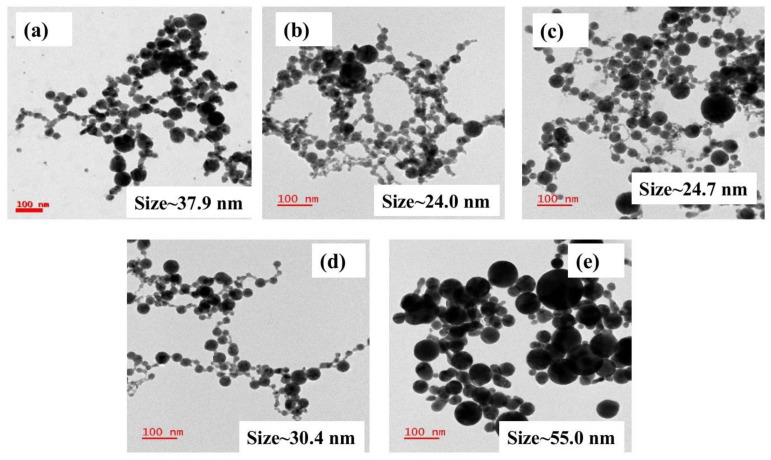
TEM images of as-fabricated NPs by laser ablation (**a**) Ag NPs (**b**) Ag_70_ Au_30_ NPs (**c**) Ag_50_Au_50_ NPs (**d**) Ag_30_Au_70_ NPs (**e**) Au NPs, respectively.

Figure 1a shows the pictures of the various fabricated NPs in glass bottles. Figure 1b illustrates the normalized absorption spectra of as-fabricated Ag, Au, and Ag-Au colloids in DW achieved by ps laser ablation. All of the absorbance spectra were normalized using software to compare the shift in the X-axis (i.e., absorbed wavelength). The SPR peaks of as-fabricated pure and alloy NPs were located at ~410 nm, ~425 nm, ~442 nm, ~471 nm, and ~521 nm for Ag, Ag_70_Au_30_, Ag_50_Au_50_, Ag_30_Au_70_, and Au, respectively. While increasing the Au ratio in the Ag-Au combination, the SPR peak was observed to be red-shifted and these results are in good agreement with the reported studies earlier [32]. The shift in Au-Ag NPs plasmon band depends predominantly on the material fraction rather than the sizes of particles. It is noticeable from the illustrated data that the observed single plasmon peak clearly established the formation of Au-Ag alloy NPs and, conceivably, there was no core-shell NPs formation. The absorption spectra would have unveiled two plasmon bands if there were any core-shell nanostructures formation. Moreover, the plasmon bands were located in the intermediate spectral region of monometallic Ag (~410 nm) and Au (~521 nm) NPs. From Figure 1c data, a linear relationship was perceived from the plot of Ag-Au NPs plasmon band with the increase of Au mole fraction. Recently, Majhi et al. [33] have theoretically studied the SPR tuning of metal alloy nanoparticles such as Au-Ag, Au-Cu, and Ag-Cu using Garcia’s modified model. They plotted SPR position against the Au molar concentration for 10 and 20 nm sizes as well as for Ag-Au alloy NPs, and in both the cases they have noticed a linear shift of SPR peak with respect to molar concentration. In another study, Hu et al. [34] have reported the optical properties of Ag Au bimetallic nanostructure (10 nm) with discrete dipole approximation theoretical simulations, which also divulged a linear relationship between SPR position and metal mole fraction. Their simulation results demonstrated that the SPR peak shifted from 415 nm (pure Ag) to 537 nm (pure Au). Figure 1c clearly demonstrates that the Ag-Au alloy NPs plasmon peak can be tweaked effortlessly by simply altering the Au molar content in the composition [35,36]. An aliquot (3 µL) of obtained colloids was deposited on a carbon-coated Cu grid, were dried at ambient conditions and, subsequently, utilized for TEM measurements. The shape, size, and crystallinity nature of the NPs were inspected by analyzing the TEM data. The as-fabricated pure and alloy NPs had spherical nature and their diameters ranged from ~10 nm to ~100 nm, which is manifested in the TEM images in Figure 2a–e). The average NPs sizes were assessed by counting >200 particles using ImageJ software. The NPs mean sizes were found to be ~37.9 nm, ~24.0 nm, ~24.7 nm, ~30.4 nm, and ~55.0 nm for Ag, Ag_70_Au_30_, Ag_50_Au_50_, Ag_30_Au_70_, and Au, respectively and the data were presented as size distribution histograms in Figure S1(a2–e2) of Supporting Information.

The mean sizes of NPs were found to be different for different Ag-Au ratios, including pure targets though the ablation experiments were conducted under similar conditions [37,38]. This could be ascribed to the difference in ablation thresholds of alloy targets as compared to the pure (Ag/Au) targets. This may have resulted in the variation of ablation yields/density as well as size variation when they ablated (pure metals/alloys) in liquid media [39,40]. In one of the previous studies, Manjón et al. [38] experimentally demonstrated that the yield of ablation was low for Ag NPs as compared to that of Au-Ag NPs when the pure (Ag, Au) and alloy (Ag-Au) targets were ablated in a polymer solution (methyl methacrylate) using 7 ps laser pulses. They anticipated that the discrepancy in the NPs size distributions was due to the dissimilar ablation thresholds of individual Au, Ag, and Ag-Au alloy targets. In our work, more interestingly, nano-chains such as morphology were evident from the TEM images of the fabricated NPs [31,32]. The probable reason could be due to the engendered high-pressure plasma at the target-water interface through the laser ablation, which can induce strong interactions between the ejected material in the form of plasma from the target surface and the surrounding liquid. Further, laser sintering, as well as collision-induced aggregation among the NPs, could also have favored the formation of such structures. Several authors have recently demonstrated the formation of nano chains-like structures when the ablation was performed in water and precursor (HAuCl_4_) solutions and they proposed that the self-assembly of NPs might lead to the observation of such nanochains.

**Figure 3:**
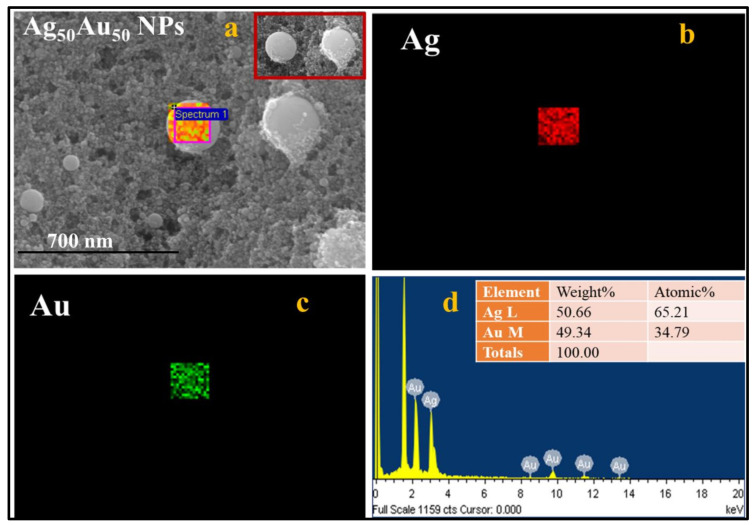
(**a**) FESEM-EDX map image of a Ag_50_ Au_50_ particle. The highlighted portion was utilized to collect the EDX data while the inset shows the two nanoparticles (**b**) Ag EDX map (**c**) Au EDX map (**b**) EDX spectra of Ag_50_ Au_50_ NP. The scale bar for (**a**–**c**) is 700 nm. The inset of (**d**) shows the respective compositions of Ag and Au in a single Ag_50_Au_50_ particle.

Ag-Au alloy NPs formation was confirmed by performing the energy dispersive x-ray (EDX) mapping and local analysis in a square area on Ag_50_Au_50_ particle, which is depicted in Figure 3a. The presented color map image revealed that the single-particle was composed of both the atoms, Ag and Au. As shown in Figure 3a mixed color map, the individual Ag and Au atoms are represented in red and green colors, respectively, and are shown in Figure 3b,c, respectively. The data presented in Figure 3d demonstrates the weight percentages of Ag and Au atoms being 50.66 and 49.34, respectively, in a selected region of Figure 3a, which is compatible with the ratio of Ag and Au in Ag_50_Au_50_ bulk strip. The mechanism of alloy NPs formation can be described as follows. When a high-intensity laser pulse interacts with the Ag-Au target, it produces high-temperature plasma by absorbing the input laser pulse energy, and as a consequence, ejected atoms from the target surface vaporize, thereby leading to the formation of dense Ag and Au atoms in the vicinity of the laser spot. Moreover, the interaction between the ejected metal atoms is much stronger than the interaction between the metal and the surrounding liquid. The fashioned metal atoms will tend to aggregate and generate NPs due to the formation of solid solutions [41] (Ag and Au constituents are homogeneously and randomly mixed at the atomic level) of Ag and Au at all compositions. Furthermore, the mixing process of Ag and Au is thermodynamically favorable due to their similar lattice parameters. Therefore, the formed NPs will be homogeneous bimetallic/alloy NPs. The results obtained from the fabrication of Ag-Au NPs are consistent with the LAL studies demonstrated earlier [37]. The distribution of NPs onto the filter paper, as well as their elemental compositions, were investigated by collecting the data from the FESEM-EDX instrument.

**Figure 4:**
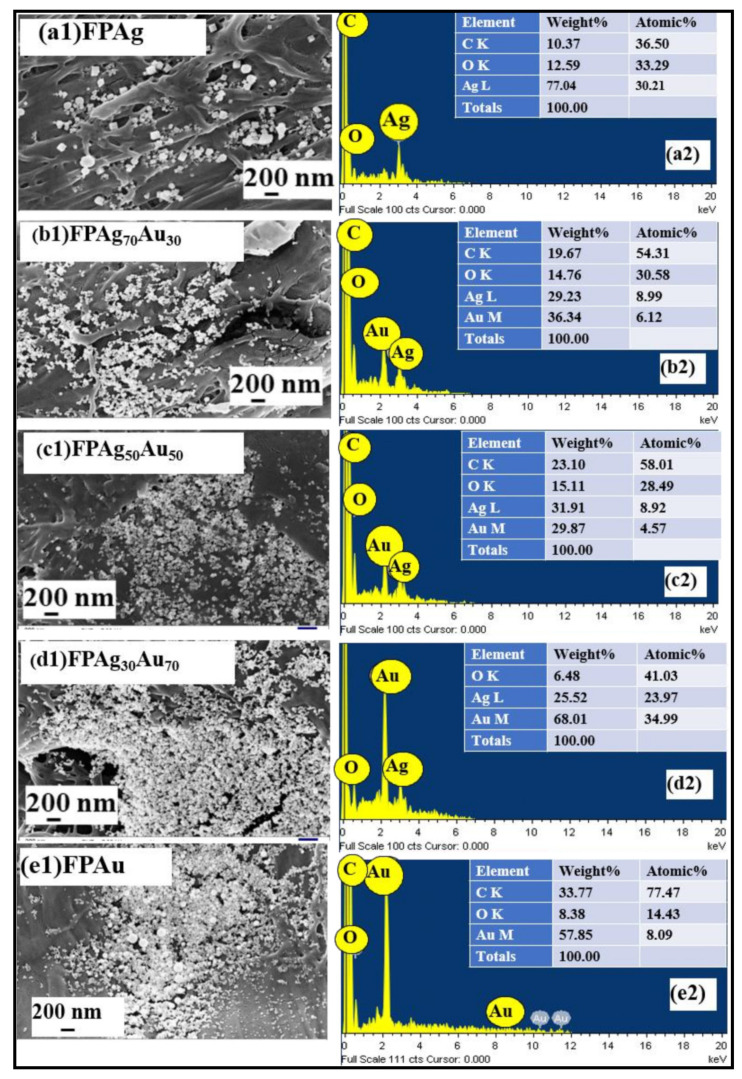
FESEM images of filter paper-loaded pure and alloy NPs and their corresponding EDX spectra of (**a1**,**a2**) Pure Ag, (**b1**,**b2**) Ag_70_ Au_30_, (**c1**,**c2**) Au_50_Ag_50_, (**d1**,**d2**) Ag_30_Au_70_, and (**e1**,**e2**) Pure Au, respectively.

Figure 4 illustrates the FESEM micrographs and their corresponding EDX images of FP-loaded laser-fabricated NPs. Figure 4a1,a2 depict the FESEM image of Ag NPs-loaded FP and its corresponding EDX, revealing the presence of Ag along with carbon and oxygen elements [42]. The FESEM image of Ag_70_Au_30_ NPs-loaded FP revealed a random distribution of the NPs throughout the FP and their EDX spectra confirmed the occurrence of both Ag and Au along with other elements, shown in Figure 4(b2). Similarly, FESEM images of Ag_50_Au_50_, Ag_30_Au_70_ NPs and Au NPs-loaded FP are shown in Figure 4(c1–e1), respectively. The elemental composition of Ag_50_Au_50_ NPs, Ag_30_Au_70,_ and Au NPs-loaded FP presented in EDX images, depicted in Figure 4(c2–e2), confirmed the presence of Ag and Au with different weight/atomic percentages. In all of the EDX measurements, the occurrence of C and O elements are possibly from the fiber-like morphology of the FP substrate.

## 4. SERS Studies-Pesticide, Dye and Explosive Molecules

**Figure 5:**
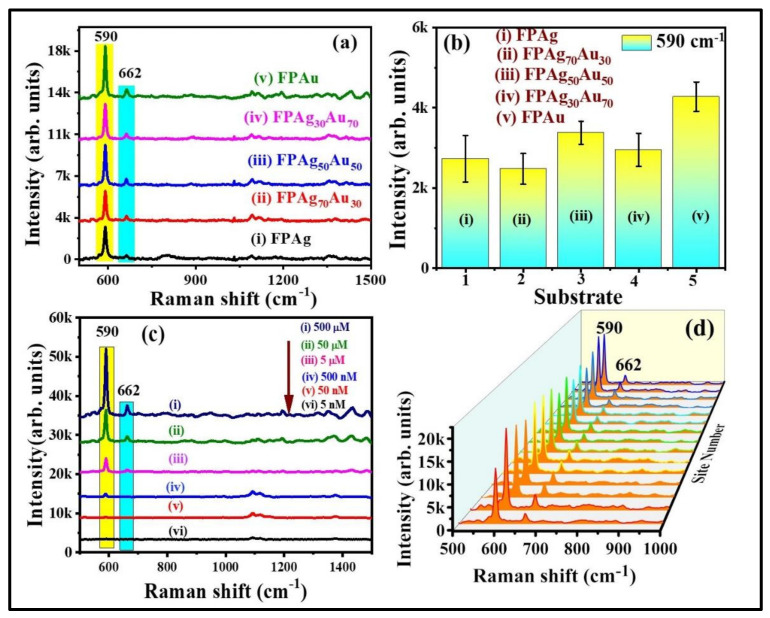
(**a**) SERS spectra of NB (5 µM) recorded from (i) FPAg (ii) FPAg_70_Au_30_ (iii) FPAg_50_Au_50_ (iv) FPAg_30_Au_70_ (v) FPAu substrates. (**b**) Histogram plot of SERS signal intensity at 590 cm^−1^ modes recorded on each substrate. Integration time is 15 s. Errors bars were estimated by measuring the standard deviation of 590 cm^−1^ peak SERS average intensity obtained from five repetitive SERS measurements on each substrate. (**c**) SERS signals of NB at varying concentrations [from (i) 500 µM to (vi) 5 nM] (**d**) 3D SERS spectra of NB (500 µM) collected from randomly selected 15 sites on FPAu substrate. Integration time was 15 s.

Further, SERS investigations were accomplished for NB and thiram detection using FP-loaded with NPs having different compositions (Ag, Ag_70_Au_30_, Ag_50_Au_50_, Ag_30_Au_70_, Au). Initially, FP-loaded pure and alloy NPs SERS activity was evaluated using NB as a probe molecule. Figure 5a,b shows the SERS spectra of NB (5 µM) and intensity variation of 590 cm^−1^ peak which is assigned to C–C–C and C–N–C deformations (All of the peak assignments are tabulated in Table S1) [43] obtained from FP loaded Ag, Ag_70_Au_30_, Ag_50_Au_50_, Ag_30_Au_70_, and Au NPs substrates. Among all of the substrates, the FPAu substrate has depicted a superior enhancement, which could be ascribed to the multiple reasons such as (i) Au NPs had a greater mean size (~55 nm) among the other compositions including pure Ag NPs which could influence the SERS performance; (ii) a large number of loaded NPs onto the filter paper (Au NPs density was greater, which can be observed from the photographic color images of NPs even though we have not measured the density exactly) leading to the formation of more hotspots resulting in prominent SERS effect; (iii) Plasmonic band of the Au NPs was red shifted after loading onto the filter paper (data are not shown) which is closer to the excitation wavelength (785 nm) of the utilized Raman spectrometer and this could also possibly affect the SERS capability. Previously, Oscar et al. [44] have demonstrated the synthesis of Ag, Au, and Au-Ag alloy NPs from the ablation of bulk alloy targets in ethanol using ns laser pulses. Further, they utilized the pure and alloy NPs coated on silicon/glass substrate for MB detection and found the high EFs for MB with Ag NPs in comparison with the other combinations, even though the mean size of Ag NPs (~11 nm) was lower than the others (~15–23 nm). They proposed that SERS effect is not only dependent on the shape/sizes but also on the composition of particles contributing different electric fields. In another study, Fan et al. [45] investigated the SERS competence of Au-Ag bimetallic (varying composition) NPs along with the monometallic NPs for sensing of Nile blue and oxazine. They found that alloy NPs those having the higher Au molar content exhibited the strongest SERS signal. For other analytes thiophenol (TP) and 4-hydroxythiophenol (HTP), the pre-eminent SERS activity was found for those having the higher Ag content. Finally, they proposed that the enhancement of Raman signals from the alloy/pure NPs not only depends on the Ag/Au molar ratio of the Alloy NPs but also depends on the chemical nature of the analyte molecule. However, we need more systemic investigations to comment on exact parameter which plays a significant role in observed prominent SERS performance for Au NPs loaded FP substrate.

**Figure 6:**
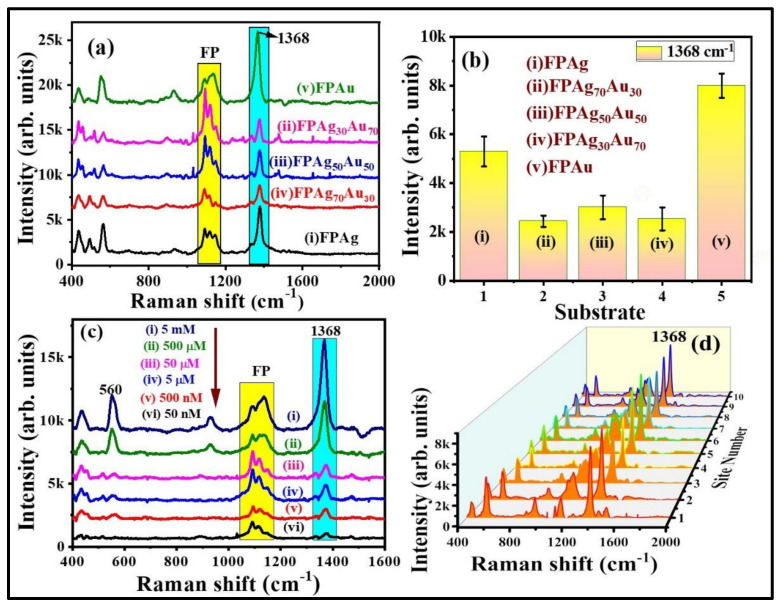
(**a**) SERS spectra of thiram (5 mM) collected from (i) FPAg (ii) FPAg_70_Au_30_ (iii) FPAg_50_Au_50_ (iv) FPAg_30_Au_70_ (v) FPAu substrates. (**b**) Histogram plot of SERS signal intensity at 1368 cm^−1^ peak recorded on each substrate. Integration time is 15 s. Errors bars were achieved by estimating the standard deviation of 1368 cm^−1^ peak SERS average intensity obtained from five repetitive SERS measurements on each substrate. (**c**) SERS signals of thiram at different concentrations (from 5 mM to 50 nM). (**d**) 3D SERS spectra of thiram (5 mM) collected from randomly selected 10 sites on FPAu substrate. Integration time was 15 s.

The SERS signals of NB at different concentrations, from 500 µM to 5 nM, were obtained on FPAu substrate to evaluate their sensitivity and the data are depicted in Figure 5c. The SERS peaks intensity decreased upon a decrease of NB concentration. The FPAu substrate had exhibited the lowest detection limit of ~10^−9^ M for NB. Figure S2a illustrates a linear plot between the NB SERS signal intensity versus concentration, and the correlation coefficient (R^2^) was observed to be ~0.95, demonstrating the detection sensitivity of the FPAu substrate. Further, SERS signal homogeneity of FPAu substrate was examined by recording the Raman spectra of NB (500 µM) at 15 random sites over the 1 cm^2^ area, and the data were presented in Figure S2b. The relative standard deviation (RSD) values for the SERS intensity at 590 cm^−1^ and 662 cm^−1^ were estimated to be 13.12% and 15.32% acquired from the FPAu substrate. Furthermore, the SERS activity of FP-loaded pure and alloy NPs was investigated with another probe molecule, which was a pesticide (thiram). Figure 6a shows the recorded SERS spectra of thiram (5 mM) from FPAg, FPAg_70_Au_30_, FPAu_50_Ag_50_, FPAg_30_Au_70,_ and FPAu substrates, respectively. As depicted in Figure 6a, the Raman peak of all SERS spectra were consistent, and they all had a prominent peak at 1368 cm^−1^ (CN stretching and symmetric deformation CH_3_) [46], along with the FP peaks [47], highlighted with blue and yellow boxes, respectively, in Figure 6a. The observed Raman peaks and their assignments of thiram are summarized in Table S2 (Supplementary Materials). The intensity variation in SERS signals acquired from each substrate is presented as a histogram plot in Figure 6b. In the case of thiram also, a prominent SERS effect was noticed from the FPAu substrate as compared to the remaining substrates.

The SERS spectra of thiram at different concentrations, ranging from 5 mM to 50 nM, were obtained on FP Au substrate, and the data are depicted in Figure 6c. The SERS intensity of thiram characteristic modes increased as the concentration increased. When the concentration of thiram was as low as 10^−8^ M, the prominent Raman mode at 1368 cm^−1^ was still predictable. There was a linear relationship between the intensity of the major peak (1368 cm^−1^) and the concentration of thiram (R^2^ = 0.88), revealing that the FPAu substrate had notable sensitivity, which was depicted in Figure S3a. Further, the SERS signal reproducibility of the FPAu substrate was assessed by considering the SERS spectra of thiram (5 mM) at randomly selected 10 spots and the data were shown as a 3D waterfall curve in Figure 6d. Intensity variation of thiram (5 mM) SERS spectra collected from 10 different sites from FPAu substrate shown as histogram plot by considering the peak intensity at 1368 cm^−1^, data depicted in Figure 6d and Figure S3b. The RSDs of Raman peak at 1368 cm^−1^ was calculated to be 10.84%, inset of Figure S3b, indicating that the FPAu substrate had a decent reproducibility.

**Figure 7:**
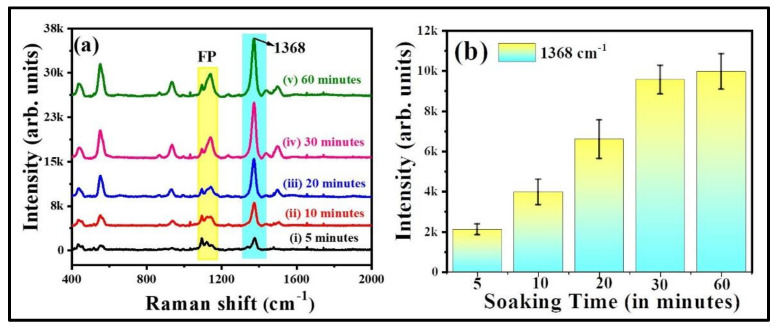
(**a**) Soaking time of filter paper in Au NPs (**b**) relative intensity of thiram (50 µM) at 1368 cm^−1^ with respect to FP soaking time. Error bars are measured based on the standard deviation of the average SERS intensity at 1368 cm^−1^ peak accomplished by repetitive SERS measurements five times. Integration time is 30 s.

**Figure 8:**
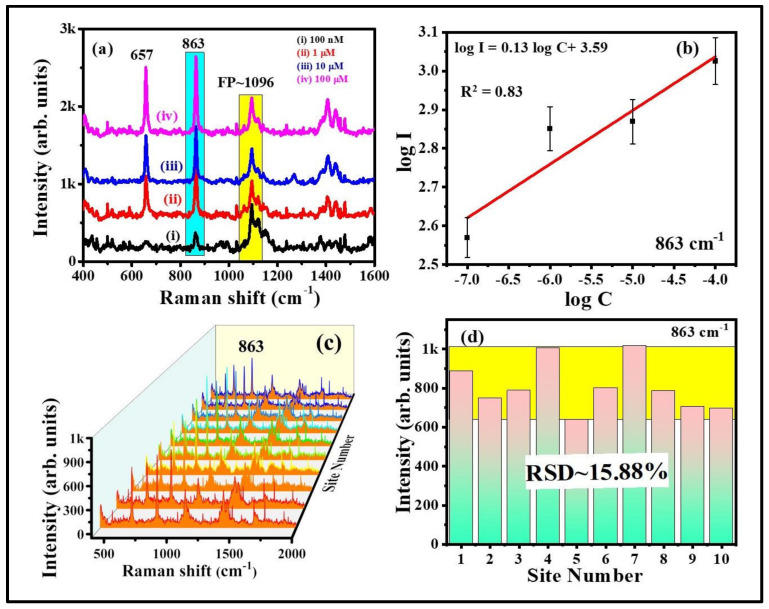
RDX SERS spectra recorded from optimized FP Au SERS substrate (**a**) concentration RDX SERS spectra (**b**) variation of SERS intensity versus concentration of the analyte (**c**) 3D waterfall SERS spectra (**d**) Histogram plot of RDX SERS signal intensity at 863 cm^−1^ peaks collected at 10 random sites.

The characteristic peak of thiram located at 1368 cm^−1^ was opted to further demonstrate the intensity changes in SERS spectra with soaking time, shown in Figure 7a. As illustrated in Figure 7b, the intensity of thiram at 1368 cm^−1^ mode was increased while increasing the soaking time of FP. The intensity enhancements were not much different at 30 and 60 min of soaking time as compared to the others. As the FP soaking time increases, the particles loading also increases onto the FP, which leads to the generation of more hotspots, resulting in a prominent SERS effect at a particular soaking time. From our experimental observations, we firmly believe that the FP soaking time of 30 min is adequate for conducting the SERS measurements. Additionally, the effect of baser paper on SERS efficiency was evaluated by soaking the commercially available sandpaper, printing paper, and FP with Au NPs for the detection of NB (50 µM), and data are presented in Figure S4a. The characteristic peak of NB located at 590 cm^−1^ was opted to further demonstrate the intensity changes in SERS spectra with respect to the substrate, shown in Figure S4b. The higher SERS performance attained for the FP-loaded Au NPs could be attributed to the porosity and fiber network-like structure of FP among the other paper substrates. Owing to the higher enhancements with excellent reproducibility, the Au NPs loaded FP substrate was further opted to perform the Raman studies for explosive (RDX). The recorded SERS signals for the RDX at different concentration is depicted in Figure 8a. The major peak of RDX SERS spectrum was located at 863 cm^−1^ and is assigned to the C–N stretching [48,49]. The observed Raman peaks and their assignments of RDX are tabulated in Table S3 (Supporting Information). The SERS intensity of RDX characteristic modes increased as the concentration increased and the data are shown in Figure 8b. When the concentration of RDX was as low as 100 nM, the prominent Raman mode at 863 cm^−1^ was still documented. There was a linear relationship between prominent Raman peak intensity at 863 cm^−1^ and concentration of RDX (R^2^ = 0.83), demonstrating that the FPAu substrate had notable sensitivity, presented in Figure 8b. The SERS signal reproducibility of the FPAu substrate was evaluated by considering the SERS spectra of RDX at randomly selected 10 spots, and the data were shown as a 3D waterfall curve in Figure 8c. Intensity variation of RDX SERS spectra collected from 10 different sites from FPAu substrate shown as histogram plot by considering the peak intensity at 863 cm^−1^, data depicted in Figure 8d. The RSDs of Raman peak at 863 cm^−1^ was calculated to be 15.88%, inset of Figure 8d, indicating that the FPAu substrate had a good reproducibility. The efficacy of the optimized FPAu substrate was estimated by calculating enhancement factors (EF) for the all of the molecules inspected, and the obtained EFs are in the order of ~10^5^. The detailed calculations are provided in the Table S4.

### Swabbing Application of the FPAu SERS Substrate

**Figure 9:**
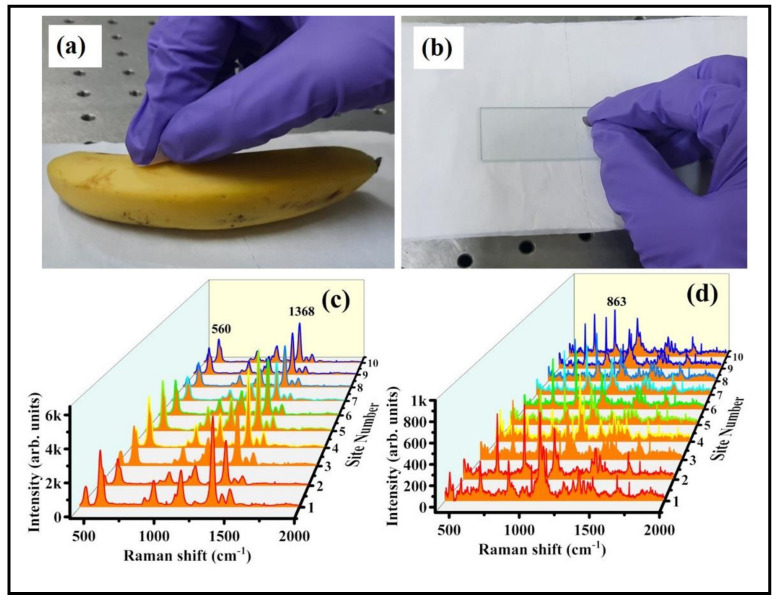
(**a**,**b**) Photograph of thiram and RDX collection by simple swabbing on banana and glass surfaces using flexible FP substrate. (**c**) Thiram SERS spectra were recorded by swabbing on a banana (**d**) RDX SERS spectra recorded by swabbing on glass and collected the data from randomly selected 10 sites on optimized FPAu SERS substrate (after swabbing).

The foremost advantage of flexible SERS substrate is the easy sample collection on real-life surfaces [50,51]. In the presented results indicated that the FPAu substrate possessed superior sensitivity and reproducibility, which could be significant for the swab sampling test. The optimized 20 min-soaked FP-Au SERS substrate was further utilized to perform the Raman studies for detection of residues (thiram, RDX) on two different surfaces to demonstrate their efficacy for real-field applications. For swabbing test, the desired analyte solution of 5 µL was drop costed using micro-pipette on a cleaned banana/glass slide followed by drying at room temperature. The photographs shown in Figure 9a,b were taken during the real-time sample collection from a banana purchased from a local fruit shop and a glass slide containing residual RDX used in lab needs. The SERS signals of thiram-50 µM (60 ng in 5 µL analyte) were obtained on a banana surface by swabbing with FPAu substrate, and the data are shown in Figure 9c. Similarly, RDX (10 mM; 11 µg in 5 µL analyte) was also detected on glass substrate by swabbing approach, data of which is presented in Figure 9d. Therefore, the lowest detectable amount of Thiram and RDX were estimated to be 60 ng and 11 µg, respectively, using the SERS swabbing test. Recently, Khan et al. [52] reported in-situ growth FP-alloy SERS substrates and utilized for the detection of Brilliant Cresyl Blue (BCB), crystal violet (CV), and R6G molecules by simple solution drying and swabbing. Gong et al. [53] have demonstrated the detection of 2,4 DNT using Ag NPs loaded cotton swab and the sample was collected from glass surface by swabbing approach. Although we have tested FPAu SERS substrate on these two surfaces (banana and glass) are limited in this work, as a proof of concept, we strongly believe that the combination of the laser fabricated NPS loaded with flexible substrate is a preeminent approach for easy sample collection along with the portable Raman spectrometer utilization for on-filed applications strengthen the significance of this work. Moreover, comparison studies on SERS performance of filter paper loaded with metal and alloy NPs including present study is provided in the supporting information (Table S5).

**Figure 10:**
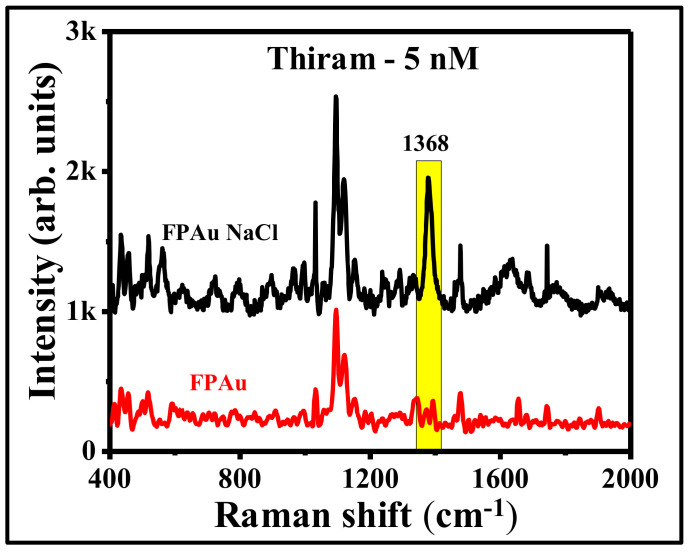
SERS spectra of thiram-5 nM molecule using FP-loaded with ps laser ablated of Au NPs in the presence of distilled water (FPAu-red color) and NaCl-20 mM (FPAu NaCl-black).

In an attempt to further improve the SERS efficiency of FP Au flexible substrate, instead of using pure Au NPs (ablated in DW), we have utilized the laser fabricated NPs in the presence of NaCl (20 mM) solution (remaining experimental conditions were kept similar). Picosecond laser ablated Au NPs in 20 mM NaCl were also found to be in spherical shape, which was confirmed from the corresponding TEM images, as shown in Figure S5a. Figure S5b,c represent the lower and higher magnification images of soaked FP in Au NPs with NaCl. Previously, [42] we have demonstrated the aggregation effect due to the presence of NaCl and a systematic study was performed further to optimize the NaCl concentration in order to achieve the prominent SERS effect. Here also, the SERS efficiency of FP loaded Au NPs with NaCl (20 mM) has demonstrated a further improvement in the detection limits of thiram. Figure 10 shows the SERS spectra of thiram 5 nM using FPAu with NaCl (20 mM) and without NaCl. Interestingly, the detection limit of thiram molecule was improved from 50 nM to 5 nM with the simple addition of NaCl during laser ablation. The addition of NaCl while ablating the targets for the production of NPs has shown an additional path to improve the SERS signals further and can be applied for on-field sample detection. The present study is significant since picosecond ablation in liquids is known to provide higher yields of the NPs compared to femtosecond LAL. Further, ps lasers are more robust, rugged, and low-cost compared to femtosecond lasers paving way for commercialization in the future. Since there are several parameters involved in LAL (pulse duration, energy, wavelength, etc.) it is imperative to investigate all of these methodologies on different analytes for accomplishing cost-effective and flexible SERS substrates for niche applications.

## 5. Conclusions

In this work, flexible FP-based SERS substrates were fabricated using a combination of individual Ag, Au and, alloy/bimetallic NPs loading onto a Whatman filter paper, and these flexible substrates were exploited for trace level detection of pesticides and explosives both in the laboratory as well as in real-time applications. The pure Au, Ag, and Au-Ag alloy nanomaterials (NPs and NSs) were prepared by submerging bulk (Au-Ag) targets in DW with various compositions Ag, Ag_70_ Au_30_, Ag_50_ Au_50_, Ag_30_ Au_70_, Au, and followed by ps pulsed laser ablation. A shift in the SPR was observed in the UV-Visible absorption spectra with varying Au proportions, which confirmed the formation of alloy NPs. Both Au-Ag alloy NPs were loaded onto the filter paper and utilized as SERS sensors for detecting thiram and Nile blue. Better SERS performance was attained for FP loaded Au NPs as compared to that of Ag loaded FP and other compositions. FP-loaded Au NPs exhibited superior SERS performance as compared to Ag, and Au-Ag bimetallic/alloy NPs loaded FP. The detected concentrations from FP-loaded Au NPs SERS substrate were up to ~10^−9^ M for NB, ~10^−8^ M for thiram and 10^−7^ M for RDX, respectively. For the pure Au NPs loaded FP, which has showed superior SERS response in this study, real-time detection of thiram and RDX from a banana and a glass slide was also demonstrated. In this work, all our SERS studies were performed in the non-resonant condition with a portable Raman spectrometer and 785 nm excitation. The 785 nm excitation system is beneficial since it delivers a better signal-to-noise ratio in the Raman signals due to lower fluorescence background in comparison with 532 nm and 632 nm laser excitations. The portable Raman spectrometer utilized here is a low-cost spectrometer (compared to a bulk micro-Raman spectrometer) and can be carried for on-site detection as compared to the laboratory-based Raman spectrometers. These kinds of plasmonic NPs loaded flexible FP-based SERS substrates render a cost effective, easy, and real-time detection of hazardous/dangerous molecules up to trace levels. Hence, this study may be helpful for further exploration in this direction.

## Data Availability

All of the data generated from this work are presented in the manuscript.

## Supplementary Materials

The following supporting information can be downloaded at: https://www.mdpi.com/article/10.3390/nano12132150/s1, Figure S1. The size histograms of fabricated NPs by laser ablation (a2) Ag NPs (b2) Ag_70_ Au_30_ NPs (c2) Ag_50_Au_50_ NPs (d2) Ag_30_Au_70_ NPs (e2) Au NPs respectively. Figure S2. (a) Logarithmic plot between the SERS intensity at 590 cm^−1^ peak and NB concentration (b) Histogram plot of SERS signal (NB-500 µM) intensities at 590 cm^−1^ and 663 cm^−1^ Raman modes collected at 15 random cites on FPAu substrate. Figure S3. (a) Logarithmic plot between the SERS intensity of 1368 cm^−1^ peak and NB concentration (b) Histogram plot of SERS signal (thiram −5 mM) intensity at 1368 cm^−1^ peak collected at 10 random cites on FPAu substrate. Figure S4. (a) SERS spectra of NB recorded from Au NPs soaked (i) sand paper (ii) printing paper (iii) FP (b) Variation of SERS intensity from each substrate. Figure S5. (a) TEM image of laser ablated Au NPs in NaCl solution (b and c) lower and higher magnification FESEM images of FP loaded with ps laser ablated Au NPs in 20 mM NaCl. Table S1. Raman peaks and their assignments of NB [43]. Table S2. Raman peaks and their assignments of Thiram [46]. Table S3. Raman peaks and their assignments of RDX [54]. Table S4. EF calculations on filter paper substrate soaked onto Au NPs for each analyte. Table S5. SERS performance of Filter paper substrates loaded with metal NPs and their alloy NPs. References [54,55,56,57] were cited in Supplementary Materials.
