# Supplementary material for: Picosecond Laser-Ablated Nanoparticles Loaded Filter Paper for SERS-Based Trace Detection of Thiram, 1,3,5-Trinitroperhydro-1,3,5-triazine (RDX), and Nile Blue
Source: Nanomaterials (Basel). 2022 Jun 22;12(13):2150. doi: 10.3390/nano12132150 (PMC9268529; doi:10.3390/nano12132150)
Supplement: Supplementary file 1 [file nanomaterials-12-02150-s001.zip › nanomaterials-1755391-supplementary.pdf]

nanomaterials

Article

Picosecond Laser-Ablated Nanoparticles Loaded Filter Paper for SERS-Based Trace Detection of Thiram, 1,3,5-Trinitroperhydro-1,3,5-triazine (RDX), and Nile Blue

Chandu Byram [1,†], Jagannath Rathod [1,†], Sree Satya Bharati Moram [1], Akkanaboina Mangababu [2] and Venugopal Rao Soma [1,*]

1 Advanced Centre of Research in High Energy Materials (ACRHEM), University of Hyderabad, Hyderabad 500046, India; chandubyram@gmail.com (C.B.); 19acpp04@uohyd.ac.in (J.R.); mssbharathi@uohyd.ac.in (S.S.B.M.)
2 School of Physics, University of Hyderabad, Hyderabad 500046, India; mangababuind@gmail.com
* Correspondence: soma_venu@uohyd.ac.in or soma_venu@yahoo.com; Tel.: +91-40-23138811; Fax: +91-040-23012800
† These authors contributed equally to this work.

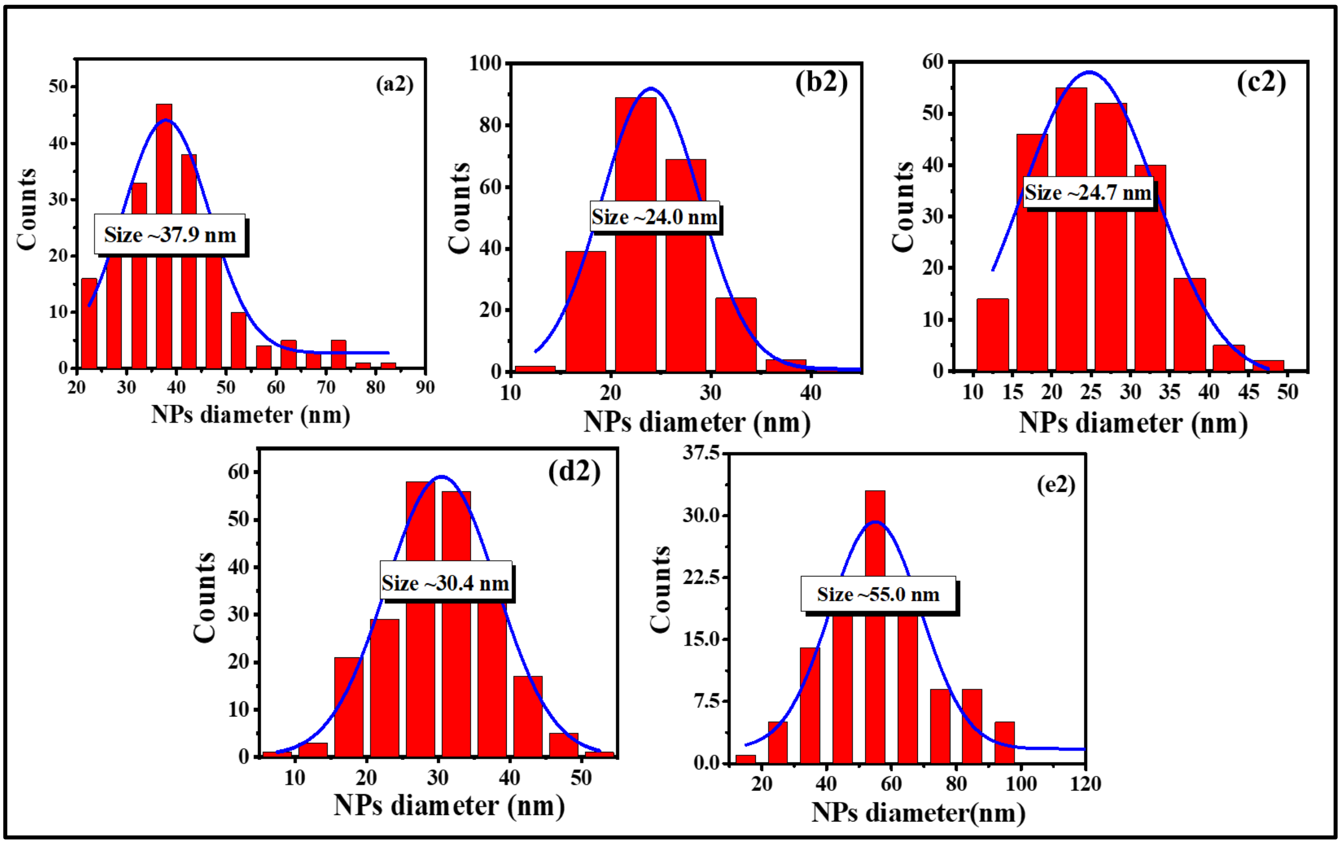

Figure S1. The size histograms of fabricated NPs by laser ablation (a2) Ag NPs (b2) Ag_{70} Au_{30} NPs (c2) $Ag_{50}Au_{50}$ NPs (d2) $Ag_{30}Au_{70}$ NPs (e2) Au NPs respectively.

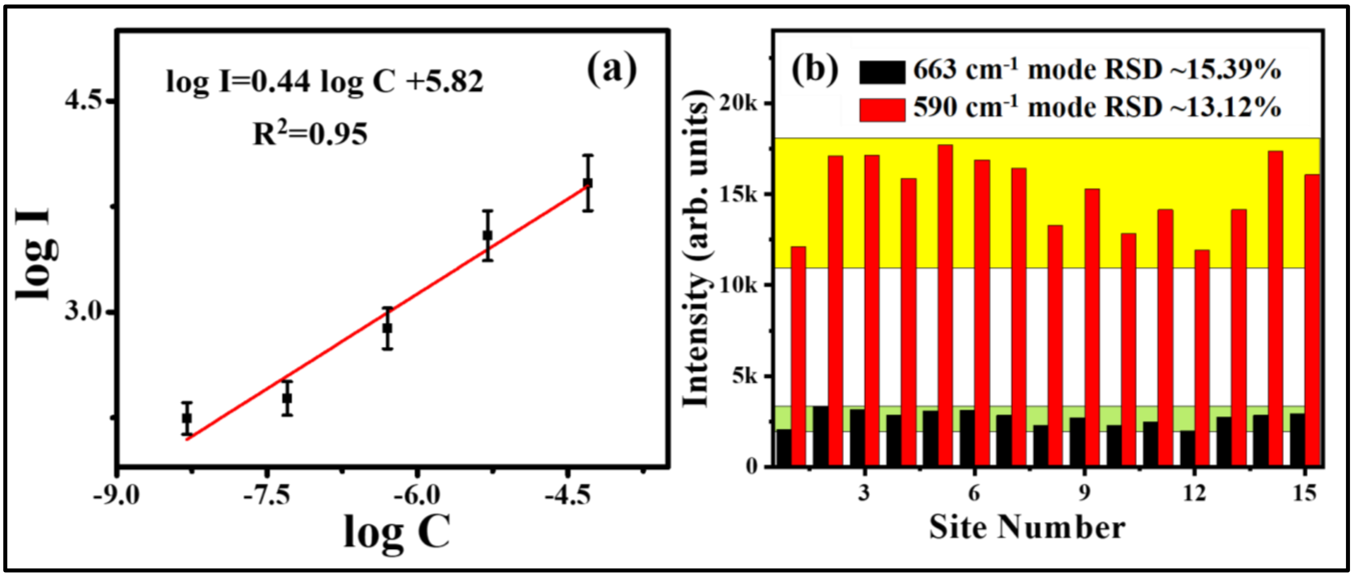

Figure S2. (a) Logarithmic plot between the SERS intensity at 590 cm^{-1} peak and NB concentration (b) Histogram plot of SERS signal (NB- 500 μM) intensities at 590 cm^{-1} and 663 cm^{-1} Raman modes collected at 15 random cites on FPAu substrate.

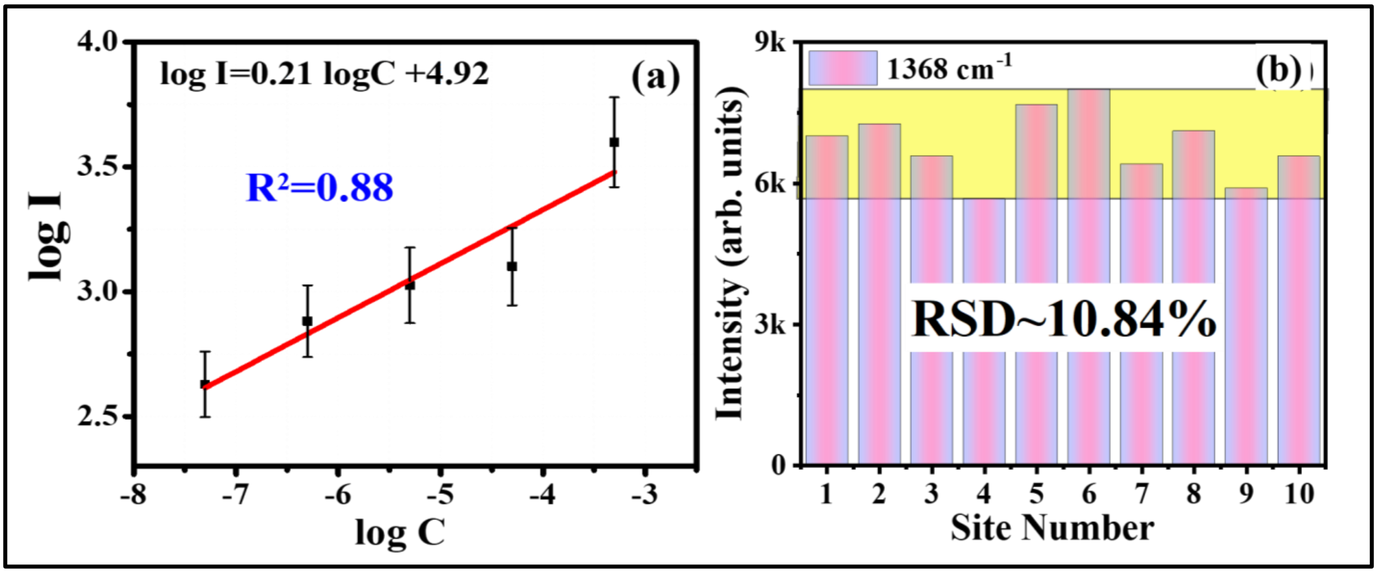

Figure S3. (a) Logarithmic plot between the SERS intensity of 1368 cm^{-1} peak and NB concentration (b) Histogram plot of SERS signal (thiram -5 mM) intensity at 1368 cm^{-1} peak collected at 10 random cites on FPAu substrate.

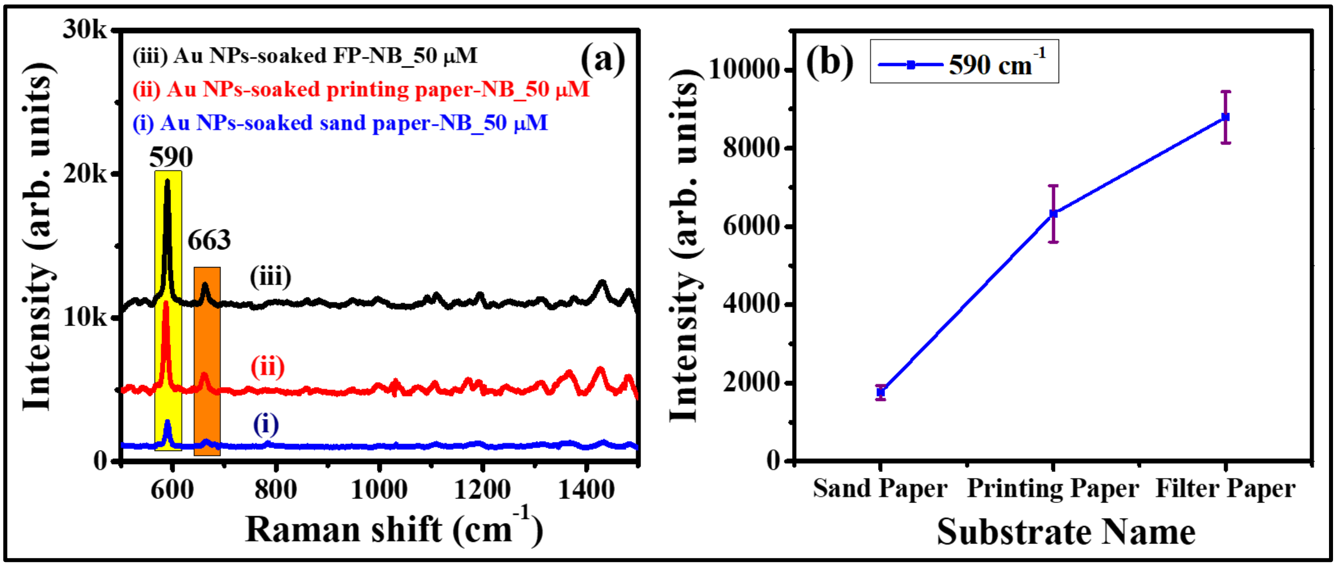

Figure S4. (a) SERS spectra of NB recorded from Au NPs soaked (i) sand paper (ii) printing paper (iii) FP (b) Variation of SERS intensity from each substrate.

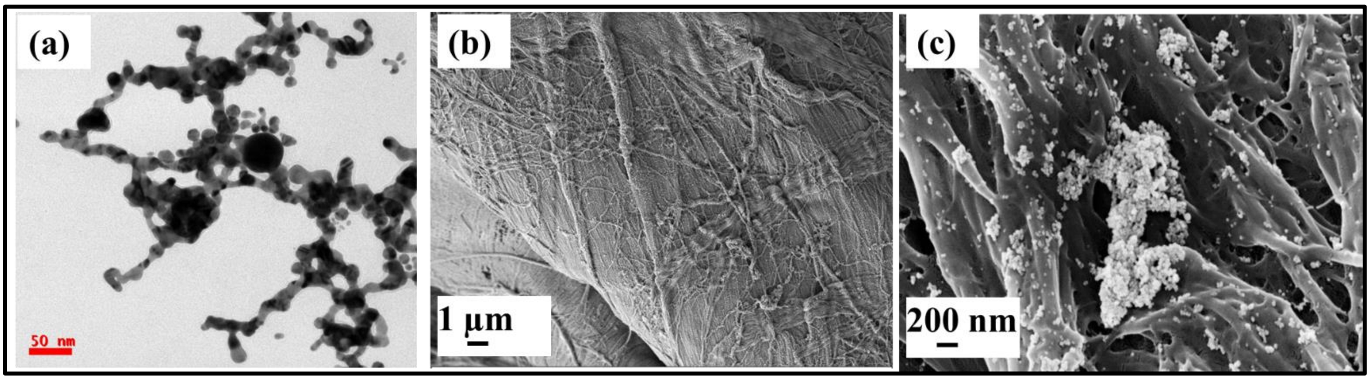

Figure S5. (a) TEM image of laser ablated Au NPs in NaCl solution (b and c) lower and higher magnification FESEM images of FP loaded with ps laser ablated Au NPs in 20 mM NaCl.

Table S1. Raman peaks and their assignments of NB [1].

S. No.	Raman shift (cm^{-1})			Peak Assignments
	Reported	Observed (powder)	Observed (SERS)	
1	499	496		C-C-C deformation
2	595	590	590	C-C-C and C-N-C deformations
3	673	663	662	In-plane CCC or NCC deformations
4	1185	1185	1118	C-H bending
5	1351,1492 and 1643	1350,1484 and 1629	1375	Ring stretching

Table S2. Raman peaks and their assignments of Thiram [2].

S. No.	Raman shift (cm^{-1})			Peak Assignments
	Reported	Observed (powder)	Observed (SERS)	
1	444	440	435	CH_3NC deformation and C-S stretching
2	559	557	560	S-S stretching
3	870	849		CH_3N stretching
4	932	973	936	C-Sand CH_3N stretching
5	1143	1148	1138	C-N stretching mode
6	1376	1373	1368	CN stretching and symmetric deformation CH_3

Table S3. Raman peaks and their assignments of RDX [3].

S. No.	Raman shift (cm^{-1})			Peak Assignments
	Reported	Observed (powder)	Observed (SERS)	
1	750		657	Ring bending with NO_2 scissoring
2	795	786		C–N stretching; NO_2 scissoring
3	850	847	-	N–N stretching; NO_2 axial scissoring
4	884	884	863	C–N stretching
5	1024	1028	-	N–C stretching; CH_2 rocking
6	1216	1214	-	N–C stretching
7	1270	1272	1268	N–N stretching; O–N–O stretching; CH_2 twisting
8	1314	1308	-	N–N stretching; CH_2 twisting; NO_2 symmetric vibration
9	1352	1387	1406	CH_2 wagging
10	1524	1562	1439	CH_2 scissoring
11	1586	1592	-	O–N–O axial stretching, NO_2 antisymmetric vibration

Enhancement factor (EF) calculations:

The efficiency of SERS active platform with respect to the non-plasmonic substrate can be estimated by enhancement factor. There are diffeent ways of calculating the EF [4,5], here we followed the formula

$$E.F = \frac{I_{SERS}}{I_{Raman}} \frac{C_{HC}}{C_{LC}}$$

Where, I_{SERS} is the intensity of prominent Raman mode of analyte obtained from the SERS substrate, I_{Raman} is the intensity of same Raman mode of analyte accomplished from filter paper (without NPs) analyte substrate, C_{LC} is the lower concentration of the analyte adsorbed on the SERS substrate, C_{HC} is the analyte high concentration adsorbed on the filter paper substrate.

Table S4. EF calculations on filter paper substrate soaked onto Au NPs for each analyte.

SERS substrate	Analyte molecule	I_{SERS}	I_{Raman}	C_{HC}	C_{LC}	EF
Filter paper soaked into picosecond laser fabricated Au NPs	Thiram	428	311	5 mM	50 nM	1.37×10^5
	NB	176	325	0.5 mM	5 nM	0.54×10^5
	RDX	243	174	10 mM	100 nM	1.3×10^5

Table S5. SERS performance of Filter paper substrates loaded with metal NPs and their alloy NPs.

S. No.	Substrates	Nanoparticles	Probe molecule	Detection limit	Ref.
1	Filter paper	Ag/Au alloy NPs	R6G<br>CV	10^{-10}M	[6]
2	Filter paper	Ag/Au NPs	TNT	10^{-7}M	[7]
3	Filter paper	Ag/Au NPs	MB<br>PA<br>DNT<br>NTO	10^{-9}M<br>10^{-6}M<br>10^{-6}M<br>10^{-6}M	[8]
4	Filter paper	AuNRs/AuNWs	R6G	10^{-9}M	[9]
4	Filter paper	Ag, Au,<br>Ag-Au alloy NPs	Thiram<br>RDX<br>NB	10^{-8}M<br>10^{-7}M<br>10^{-9}M	Present work

References

1. Bodelón, G.; Montes-García, V.; Fernández-López, C.; Pastoriza-Santos, I.; Pérez-Juste, J.; Liz-Marzán, L.M. Au@pNIPAM SERRS tags for multiplex immunophenotyping cellular receptors and imaging tumor cells. Small 2015, 11, 4149–4157. https://doi.org/10.1002/smll.201500269.
2. Zhu, J.; Liu, M.-J.; Li, J.-J.; Li, X.; Zhao, J.-W. Multi-branched gold nanostars with fractal structure for SERS detection of the pesticide thiram. *Spectrochim. Acta Part A Mol. Biomol. Spectrosc.* **2018**, *189*, 586–593. https://doi.org/10.1016/j.saa.2017.08.074.
3. Ghosh, M.; Wang, L.; Asher, S.A. Deep-ultraviolet resonance raman excitation profiles of NH_4 NO_3, PETN, TNT, HMX, and RDX. *Appl. Spectros*. **2012**, *66*, 1013–1021, doi:10.1366/12-06626.
4. Wu, R.; Jin, Q.; Storey, C.; Collins, J.; Gomard, G.; Lemmer, U.; Canham, L.; Kling, R.; Kaplan, A. Gold nanoplasmonic particles in tunable porous silicon 3D scaffolds for ultra-low concentration detection by SERS. *Nanoscale Horiz.* **2021**, *6*, 781–790. https://doi.org/10.1039/d1nh00228g.
5. Le Ru, E.C.; Blackie, E.; Meyer, M.; Etchegoin, P.G. Surface Enhanced Raman Scattering Enhancement Factors: A Comprehensive Study. *J. Phys. Chem. C* **2007**, *111*, 13794–13803, doi:10.1021/jp0687908.
6. Khan, G.A.; Demirtaş, O.Ö.; Bek, A.; Bhatti, A.S.; Ahmed, W. Facile fabrication of Au-Ag alloy nanoparticles on filter paper: Application in SERS based swab detection and multiplexing. *Vib. Spectrosc.* **2022**, *120*, 103359. https://doi.org/10.1016/j.vibspec.2022.103359.
7. Zapata, F., López-López, M. and García-Ruiz, C.,. Detection and identification of explosives by surface enhanced Raman scattering. Appl. Spectrosc. Rev. **2016**, *51*(3), pp.227–262.
8. Moram, S.S.B.; Byram, C.; Shibu, S.N.; Chilukamarri, B.M.; Soma, V.R. Ag/Au Nanoparticle-Loaded Paper-Based Versatile Surface-Enhanced Raman Spectroscopy Substrates for Multiple Explosives Detection. *ACS Omega* **2018**, *3*, 8190–8201. https://doi.org/10.1021/acsomega.8b01318.
9. Xu, K., Zhou, R., Takei, K. and Hong, M.,. Toward flexible surface-enhanced Raman scattering (SERS) sensors for point-of-care diagnostics. *Adv. Sci.* **2019**, *6*(16), p.1900925.
